# Breastfeeding and infant gut microbiota: influence of bioactive components

**DOI:** 10.1080/19490976.2024.2446403

**Published:** 2024-12-27

**Authors:** Dongqing Xu, Fengyi Wan

**Affiliations:** a Department of Biochemistry and Molecular Biology, Bloomberg School of Public Health, Johns Hopkins University, Baltimore, MD, USA; b Department of Molecular Microbiology and Immunology, Bloomberg School of Public Health, Johns Hopkins University, Baltimore, MD, USA; c Sidney Kimmel Comprehensive Cancer Center, Johns Hopkins University, Baltimore, MD, USA

**Keywords:** Breastfeeding, gut microbiota, antibodies, complement system, maternal milk

## Abstract

Establishment of the gut microbiota during infancy is critical for host health with long-lasting implications. In this orchestrated process, microbial assembly is influenced by an increasing number of genetic and environmental factors, among which breastfeeding is considered as one of the most significant drivers for infant gut microbiota development. As the optimal diet for the infants, maternal milk provides numerous nutritional, microbial, and bioactive components to ensure the most adequate microbial growth and development of a ‘healthy’ gut microbiota during early life. This review will summarize the available evidence, particularly the recent progress, on how various bioactive compounds supplied in maternal milk affect the establishment of early-life gut microbiota during infancy and promote further interest and investigation in this important area.

## 1. Introduction

The development of gut commensal microbiota is a complex process, which is traditionally regarded as starting at birth and having a longstanding impact on host health. It is broadly believed that infancy is a critical window for establishment of the gut microbiota, during which microbial assembly evolves from being nearly sterile to a complex and diverse community.^1^ A growing body of evidence indicates that gut dysbiosis during early life precedes the development of multiple atopic diseases including asthma, atopic dermatitis, food allergy, diabetes, inflammatory bowel disease, and obesity, further underscoring infancy as a crucial period for shaping microbial structure in the gut. Among the perinatal factors that influence microbial colonization process resulting in specialized microbial ecosystems in the gut, the infant diet, especially maternal milk, has been acknowledged as one of the most preponderant elements for establishment of the infant gut microbiota.^2–4^ In particular, numerous studies on the gut microbiota compositions between breastfed and formula-fed infants underline the impact of breastfeeding on the infant gut microbiota development.^3,5,6^ In a simplified model of gut microbiota succession during infancy, the gut is initially colonized by facultative anaerobes, such as *Staphylococcus*, *Streptococcus*, *Enterobacteriaceae*, and *Lactobacillus*; these pioneering taxa create a suitable environment for succession of obligate anaerobes, such as *Bifidobacterium*, *Clostridium*, and *Bacteroides*.^3,6,7^

Breast milk has been acknowledged as the best source of nutrition for the infant, ensuring optimal growth not only during early life but also supporting health through the entire lifespan.^8,9^ Research on the impact of maternal milk on infant health dates back to 1932, in which breastfeeding was shown to be associated with lower incidence of morbidity and mortality during the first year of life.^10^ A substantial number of studies since then have consistently shown that breastfed infants experience reduced disease incidence or severity, particularly in diarrheal diseases and gastrointestinal infections, in comparison with those not receiving any breast milk.^11–15^ Of note, beyond its nutritional properties essential for infant development, maternal milk has evolved over time to provide a variety of bioactive components, which are defined as elements that “affect biological processes or substrates and hence have an impact on body function or condition and ultimately health”.^16^ Generally speaking, the infants receiving exclusive breastfeeding harbor lower microbial diversity in the gut, whereas formula-fed infants have a more diverse and mature gut microbial community.^17–19^ Indeed, it has been hypothesized that the association of breastfeeding with decreased risk for infectious and inflammatory diseases is at least in part conferred through the gut microbiota in early life.^20,21^

As the optimal diet for infants, maternal milk is a complex biofluid that stimulates the most adequate microbial growth in the infant gut; numerous components present in maternal milk have been recognized to be essential for a “healthy” gut microbiota during infancy.^22^ The macronutrients (fat, proteins, and carbohydrates) and micronutrients (vitamins and minerals) supplied in maternal milk are fundamental energy sources for the growth and development of gut microbial community.^16,23–25^ Maternal milk harbors its own microbiota consisting of numerous microbes; recent studies have started to vigorously explore this rich and diverse microbial community during breastfeeding as a potential source for the microbial colonization in infant gut, which could substantially impact the establishment of infant gut microbiota.^26–28^ Beyond nutrient components and microbial species, an array of bioactive components have also been identified in maternal milk. These include lactoferrin, lysozyme, antibodies, complement components, and others, all of which are generally assumed to execute important functions in aiding organ development, facilitating immune maturation, avoiding harmful inflammation, and protecting infants against infections.^16,21,29^ Studies in human cohorts and preclinical models demonstrate that these maternal milk-supplied bioactive components evolve during the stages of lactation, most of which with relatively high concentrations in the colostrum, to guide the assembly of microbial community in the infant gut. Here we will review the available data, highlighting the critical role of various bioactive components in maternal milk on establishment of the infant gut microbiota during early life, encouraging further research in this essential area.

## 2. Oligosaccharides

Oligosaccharides are one of the major constituents of maternal milk, and the amount of milk oligosaccharides is generally higher in the early stages of lactation, with 20–25 g/L in human colostrum and 5–15 g/L in human mature milk, respectively.^30–32^ So far, over 200 different human milk oligosaccharides (HMOs) have been identified, most of which the structures are characterized. The HMOs vary in size, ranging from 3 to 22 monosaccharide units, and are primarily composed of D-glucose (Glc), D-galactose (Gal), *N*-acetylglucosamine (GlcNAc), L-fucose (Fuc), and *N*-acetylneuraminic acid (Neu5Ac or sialic acid). Typically, all HMOs contain a lactose core (Galβ1-4Glc) at their reducing end, which can be further extended through β1–3 or β1–6 linkages with repetitions of *N*-acetyl-lactosamine (LacNAc; β-D-Gal-β-(1,4)-D-GlcNAc, type 2 chain) or lacto-N-biose, (LNB; β-D-Gal-(1,3)-D-GlcNAc, type 1 chain), and these can be modified with fucose or sialic acid substitutions, leading to three categories, *i.e*., neutral (unsubstituted), fucosylated, or sialylated HMOs.^32–34^ Two key fucosyltransferases, FUT2 (encoding the secretor locus, Se) and FUT3 (encoding the Lewis blood group locus, Le), significantly influence the variability of HMOs composition.^35^ Depending on the expression of FUT2 and FUT3 in the mammary gland of individual woman, four distinct milk phenotypes can be identified: secretor positive and Lewis positive (Se+, Le+), secretor negative and Lewis positive (Se−, Le+), secretor positive and Lewis negative (Se+, Le−), and secretor negative and Lewis negative (Se−, Le−).

The gut microbiota in infants breastfed by secretor mothers (Se+, Le+ and Se+, Le−) who produce higher amounts of HMOs achieves faster establishment than in infants breastfed by non-secretor mothers (Se−, Le+ and Se−, Le−), who provide relatively lower amounts of HMOs.^36,37^ Additionally, there are subtle differences in the gut microbiota composition between infants breastfed by secretor versus non-secretor mothers, notably with higher colonization by *Bifidobacterium adolescentis* and absence of *Bifidobacterium catenulatum* in the gut of non-secretor mother-breastfed infants, underscoring an important role of HMOs in establishment of the infant gut microbiota.^38^ As documented in previous studies,^39,40^ *Bifidobacterium* species are highly specialized in utilizing HMOs; thus, breastfeeding could markedly promote the abundance of *Bifidobacterium* species in the early-life gut microbiota. The digestion of HMOs by *Bifidobacterium* produces short-chain fatty acids (SCFAs); therefore, HMOs serve as a means not only to nurture a healthy gut microbiota but also to deliver SCFAs to the infant intestines. Indeed, breastfed infants harbor higher levels of obligate anaerobes, in particular *Bifidobacterium longum*, *Bifidobacterium breve*, and *Bifidobacterium bifidum*, compared to *Bifidobacterium adolescentis*, *Bifidobacterium animalis*, and *Bifidobacterium dentium* during the first few weeks of life, largely attributed to the presence of HMOs in breast milk.^41–43^ Of note, these *Bifidobacterium* populations decline over time, while those microbes that specialize in digesting dietary fibers, *e.g*., *Bacteroides* and *Prevotella*, become more dominant as the infant gut develops.^4^

As non-digestible molecules for most commensal bacteria in the infant gut, oligosaccharides present in maternal milk intensely impact the composition of infant gut microbiota, supported by mounting evidence from human cohorts and preclinical models.^44–46^ In particular, the gut microbiota composition of breastfed infants is dramatically different from that of infants fed with formula, which lack maternal milk-supplied oligosaccharides.^36,47^ Moreover, the gut microbiota in infants breastfed by the secretor mothers (Se+, Le+ and Se+, Le−) is established faster than those by the non-secretor mothers (Se−, Le+ and Se−, Le−),^36,37^ despite the slightly different gut microbiota compositions in infants breastfed by secretor versus non-secretor mothers.^38^

Of note, besides direct consumption of HMOs by *Bifidobacterium* species, most bacteria could cleave one or two HMO linkages and work in concert with other species to completely utilize HMO substrates, known as cross-feeding.^40,48^ The cross-feeding between HMO consumers and non-HMO users in the gut plays an important function in shaping infant gut microbiota.^42^ Indeed, supplementation of 2′-FL, one of the most extensively studied synthetized HMOs, increased the abundance of *Akkermansia*, a probiotic potentially involved in the expression of mucins in goblet cells.^49^ Moreover, HMOs were reported to prevent the attachment of enteric pathogens (e.g., *Campylobacter jejuni*, *Escherichia coli, Entamoeba histolytica*, and others) to epithelial surface in the infant gut.^50–52^ Additionally, HMOs were proposed to directly bind to Toll-like receptor 4 (TLR4), hence inhibiting TLR4 signaling in *ex vivo* gut tissue and organoid cultures derived from newborn mice and premature piglets.^52^

## 3. Lactoferrin

Lactoferrin, an important whey protein in maternal milk with concentrations of 7 g/L in colostrum and 1 g/L in mature milk, respectively, possesses significant microbicidal properties.^53–55^ As a well‐established iron‐binding protein,^56,57^ the best characterized function of lactoferrin is the sequestration of iron via its high-affinity binding, thus limiting iron availability to bacteria in the gut.^58^ In addition, the iron-free form of lactoferrin exhibits direct bactericidal ability in a wide range of microorganisms including the bacteria *Streptococcus mutans, Streptococcus pneumoniae, Escherichia coli, Vibrio cholera, Pseudomonas aeruginosa* as well as fungal pathogen *Candida albicans*.^59^ Bovine lactoferrin was also reported to protect against *Rotavirus* through preventing the virus from attaching to intestinal cells and blocking a post-absorption step that is critical for new viral particle formation.^60^ Beyond its iron sequestration function, lactoferrin was proposed to inhibit bacterial growth and prevent biofilm formation in the gut, through its direct interactions with the lipopolysaccharides (LPS) that are outside of Gram-negative bacteria.^61,62^ Additionally, lactoferrin has been proposed to promote the growth of a large set of probiotic *Bifidobacterium* strains, hence maintaining intestinal homeostasis.^63^ It is noteworthy that a portion of lactoferrin could be broken down by gastric pepsin^64^ as well as at the site of infection by bacterial or mammalian proteases,^65^ to generate lactoferricin. Lactoferricin exhibits anti-bacterial properties against an array of bacteria including *Listeria*, *Escherichia coli*, *Salmonella*, and *Campylobacter*.^66^ In addition, lactoferricin has been shown to display cytotoxic activity against viruses^67^ and fungi,^68^ and prevent the attachment of bacteria to intestinal cells.^69^ Moreover, lactoferricin was proved to disrupts liposomes, leading to leakage and fusion of vesicles, which enables lactoferricin to penetrate the membrane thus interrupting cytoplasmic targets.^70,71^

The amount of lactoferrin in maternal milk varies during the stages of lactation, with a highest concentration in colostrum.^72,73^ While lactoferrin levels decrease gradually through the first month of lactation, the lactoferrin present in maternal milk is resistant to degradation by the reduced digestive capacity in the infant, thus maintaining its anti-bacterial properties to restrict bacterial growth during early life.^74^ Indeed, maternal lactoferrin was found stable in the feces of breastfed infants.^57,75^ Of note, recent clinical studies underscore the potential benefits of lactoferrin on promoting infant health. In a randomized controlled trial, the incidence of late-onset sepsis was substantially reduced in preterm infants receiving bovine lactoferrin supplementation, compared with the rate in the placebo group.^76^ Moreover, the data from recent feeding trials with at least 1,500 participants further support the potentially favorable effects of lactoferrin supplementation in preterm infants.^77^

## 4. Lysozymes and other enzymes

Lysozyme, a *N*-acetyl muramidase enzyme, is a major part of the whey protein fraction in maternal milk, particularly in colostrum.^57^ Lysozyme exhibits various bactericidal properties, which are best characterized by its lysis of Gram-positive bacteria by hydrolyzing the peptidoglycan polymers of cell walls at the 1,4-β-linkages between the *N*-acetyl-muramic acid and *N*-acetyl-d-glucosamine residues.^78^ In addition, lysozyme and lactoferrin could synergistically to clear Gram-negative bacteria, as illustrated in an *in vitro* study using electron microscopy where lactoferrin could cause the lipopolysaccharides release and facilitate lysozyme to access and degrade the peptidoglycan polymers of the bacteria, thus killing the pathogens.^79^ Moreover, lysozyme was previously reported to display anti-viral activity and the ability of lysing *Micrococcus lysodeikticus*.^80^ These findings signify the potential relevance of maternal milk-supplied lysozyme in influencing the infant gut microbiota and protecting breastfed infants from enteric pathogens.

Besides lysozyme, additional anti-bacterial proteins found in breast milk could impact the establishment of infant gut microbiota. Among them, lactoperoxidase is an enzyme secreted from the mammary gland and has been documented in human breast milk, with approximately 11 mg/mL in colostrum and 13 mg/mL in mature milk, respectively.^81^ Of note, lactoperoxidase present in maternal milk is not susceptible to degradation by pepsin and other proteases; therefore, it is most likely that the activity of ingested lactoperoxidase could survive in the infant gut with reduced digestive capacity. The anti-microbial activity of lactoperoxidase has contributed substantially to host defensive mechanisms. In particular, lactoperoxidase, in the presence of hydrogen peroxide, catalyzes the oxidation of thiocyanate to produce hypothiocyanite, an intermediary oxidation product known to decrease the viability of bacteria, viruses, and fungi.^82–84^ Additionally, additive or synergistic anti-microbial effects between lactoperoxidase and other protective factors in maternal milk, including sIgA, lactoferrin, and lysozyme, have been reported already.^85–87^

Lactadherin is a glycoprotein produced by mammary epithelial cells during lactation,^88^ and the concentration of lactadherin in human breast milk peaks immediately postpartum and then declines thereafter.^89^ Previous studies showed that lactadherin binds to Rotavirus and prevents the viral attachment to the receptors on human cell surface and that Rotavirus symptoms in the infected breastfed infants appear inversely associated with the concentrations of lactadherin.^88^ In addition, haptocorrin, a Vitamin B12-binding protein present in human milk, substantially contributes to the host defense against the growth of bacteria,^90^ hence likely playing an important function in shaping the infant gut microbiota and promoting infant health.

## 5. Cytokines and growth factors

Maternal milk contains a variety of cytokines that modulate immune system and gut microbiota composition in the infant. These cytokines are small soluble glycoproteins that act as intercellular messengers, eliciting particular responses by binding to specific receptors on target cells, which includes interleukin 1 β (IL-1β), IL-2, IL-6, IL-8, IL-10, IFN-γ, TNF-α, and others.^91^ While the primary source of these immunomodulatory cytokines is the mammary gland, leukocytes present in maternal milk also possess the capacity to secrete them.^92^ Of note, neonates harbor a limited capacity to produce some cytokines, making maternal milk an important source of cytokines during infancy. Previous research showed that certain human milk-supplied cytokines are resistant to degradation, thus remaining active in the infant gut.^93^ The precise roles of cytokines in modulating the infant gut microbiota have not been extensively investigated yet; that said, it is generally believed that cytokines act as messengers, communicating with other components in maternal milk, to modulate the development of immune system in the infant. The proposed functions include promoting the development of T cell responses, aiding in maturation of neonatal leukocytes, regulating inflammation, and facilitating production of immunoglobulins, all of which could substantially impact the composition of infant gut microbiota.^94,95^ It seems not surprising that immunomodulatory cytokines present in maternal milk participate in the establishment, development, and maintenance of gut commensal microbiota in the infant.^94,95^

Several growth factors are supplied in maternal milk, of which epidermal growth factor (EGF) plays a critical role in intestinal development,^96^ gut barrier maturation,^97^ and epithelial homeostasis.^98^ In human breast milk, EGF is most abundant in the colostrum and has previously been reported to offer protective effects against necrotizing enterocolitis,^99–102^ as well as late-onset sepsis.^103^ Multiple mechanisms have been proposed to explain EGF-mediated protective function for infant health during early life, which includes the inhibition of TLR4-mediated proinflammatory signaling pathway,^100^ the enhanced proliferation of intestinal epithelial cells,^104^ the augmented mucus production and goblet cell density,^99^ and the blocking of bacterial pathogen attachment in the gut. In addition, another EGF family member, heparin-binding EGF-like growth factor (HB-EGF), was shown to bind to certain pathogenic bacteria, thus protecting the developing gut against infection-associated intestinal injury.^105,106^

## 6. Hormones

Hormones such as leptin, adiponectin, ghrelin, insulin, resistin, obestatin, apelin and others have been identified in maternal milk, with the vast majority are transported from maternal circulation into maternal milk, whereas several are synthesized in the mammary gland.^74,107^ In general, the concentrations of hormones are higher in maternal milk than in plasma, partially attributed to the glycosylation in the mammary gland prior to secretion into maternal milk where their degradation is markedly delayed.^74^ The colostrum and transition milk contain higher concentrations of hormones, compared to those in the mature milk, which impacts the regulation of infant growth and development.^108–110^

Maternal milk-supplied hormones retain biological activity in the infant gut, where they assist intestinal cell proliferation and facilitate mucosal growth, enterocyte migration, villus development, as well as increase expression of glucose transporters.^111^ Given the known gut trophic effects of leptin, adiponectin, and insulin, their presence in maternal milk has drawn particular interest and extensive investigation in the past years.^112–115^ The contributions of hormones to the establishment of infant gut microbiota have begun to be elucidated by findings from both human cohorts and preclinical models. In a recent comprehensive study using 2-week-old exclusively breastfed infants,^116^ Lemas and colleagues reported that a positive association between insulin supplied in human breast milk and microbial taxonomic diversity as well as the abundance of Gamma-proteobacteria (*e.g*., *Enterobacteriaceae*), whereas insulin levels appear negatively associated with the abundance of *Lactobacillales* (*e.g. Streptococcaceae*). In contrast, no association was observed between leptin present in human breast milk and the infant gut microbiota composition. Moreover, metagenomic analyses revealed that leptin and insulin levels in human breast milk were correlated with decreased bacterial proteases (indicative of gut permeability) and reduced concentration of pyruvate kinase (indicative of pediatric gut inflammation).^116^ Experimental evidence in mice and rats also supports a potential role of maternal milk-supplied hormones in shaping the gut microbiota during early life. For instance, rats supplemented with leptin or adiponectin during their first 21 days of life, exhibited the presence of *Blautia*, albeit the decreased abundance of *Proteobacteria*.^117^ Supplementation with leptin in rats resulted in lower levels of *Sutterella* and augmented abundance of *Clostridium*, while supplementation with adiponectin led to lower abundance of *Roseburia* and an elevated proportion of *Enterococcus*.^117^ Moreover, experimental data generated from suckling mice that received insulin every day indicated that oral insulin supplementation may upregulate specific endotoxin receptor in the gut, thus playing an important anti-microbial function against potential pathogens.^118^

## 7. Immunoglobulins

The immunoglobulins are one of the most important bioactive components in maternal milk, which provide passive immunity to the infant and shape the infant gut microbiota. Secretory IgA (sIgA), secretory IgM (sIgM), and IgG are the most abundant immunoglobulins in maternal milk. Importantly, maternal milk is the only source of sIgA for the infant during the first four weeks of life, because infants lack functional plasma cells.^119,120^ The sIgA is crucial for the establishment of infant gut microbiota. As previously reported, the gut microbiota composition of breastfed infants substantially differs from that of formula-fed infants.^121^ Some of the sIgA is believed to be nonspecific and exhibits extensive cross-reactivity to the microorganisms in gut microbiota.^122^ Of note, the actions of sIgA appear fundamentally local, compromising the first line immune defense where sIgA binds to either commensal or pathogenic microbes, toxins, or other antigenic materials like LPS. The binding of sIgA efficiently prevent the adherence and even penetration of gut microorganisms into the epithelium, without triggering potentially harmful inflammatory responses during early life – so important a phenomenon is known as immune exclusion.^123^ For instance, sIgA in maternal milk was shown to inhibit the binding of *Clostridium difficile* toxin A to enterocyte brush border membrane receptors^124^ and was proposed to serve as a decoy receptor for enterotoxigenic *Escherichia coli*.^125^ Beyond effectively recognizing and eliminating pathogenic microbes, sIgA maintains a mutually beneficial relationship with commensal bacteria, particularly as sIgA coexists with commensal bacteria along the mucosal layer. Indeed, as much as 74% of bacteria in the gut lumen were estimated to be coated with sIgA,^126^ hence there is no surprise that sIgA plays a crucial role in guiding the assembly of infant gut microbial community. In addition, sIgA-conferred bacterial enchainment is proposed to efficiently remove invasive/inflammatory microorganisms from the microbiota, thus preventing them from acquiring new genetic components from other commensal bacteria in the gut.^127^ Moreover, sIgA could serve as a carbohydrate source and even a distinct mucosal niche to support the stable colonization of certain bacteria, *e.g*., *Bacteroides* to the epithelium.^128,129^ Accumulating evidence suggests that sIgA is crucial for shaping the composition of a healthy gut microbiota in suckling infants, by promoting the growth of obligate anaerobes (*e.g*., *Bacteroides* and *Firmicutes*) whereas limiting the proliferation of inflammatory facultative anaerobes (*e.g*., *Enterobacteriaceae*).^129,130^ It is noteworthy that distinct from IgG or IgM, the anti-bacterial activity of sIgA is not conferred by complement-mediated cytolysis or through opsonization and phagocytosis, because that sIgA binding *per se* spontaneously inhibits these complement-involved functions.^22,131^

Albeit its relatively low abundance in maternal milk, IgG has important functions in the infant gut microbiota development. IgG substantially affects the infant gut microbiota either *in utero* (since it can cross the placenta) or through maternal milk after delivery. IgG in maternal milk has also been suggested to act directly on microorganisms in the gut, which adds another dimension to the complex yet not fully understood mechanisms. In particular, mucosal IgG in mice targets mucus-consuming bacterium *Akkermansia muciniphila*, which lives close to the intestinal epithelium.^132^ While in general maternal milk-supplied IgG is believed to help counteract the infant deficiencies in opsonization and antibody-mediated cytotoxicity, it also recognizes some antigens expressed by enterotoxigenic *Escherichia coli* as well as other *Enterobacteriaceae* species produced and secreted in maternal milk.^133^ Indeed, *Enterobacteriaceae*-specific IgG present in maternal milk is crucial for neonatal mice to defense against enteric infections with *Citrobacter rodentium* and enterotoxigenic *Escherichia coli*.^134^ Moreover, in mouse models, maternal IgG antibodies were shown to be transferred through breast milk to the infant gut, where IgG directly cross-reacts with *Citrobacter rodentium*, thus conferring critical protection in neonates.^135^ In the meanwhile, maternal IgG deficiency is associated with alterations in the infant gut microbiota, characterized with elevated levels of *Porphyromonadaceae* in the colon, and augmented abundance of *Enterobacteriaceae* and *Prevotellaceae* in the small intestine, coinciding with increased IL-17A–producing cells.^136^

## 8. Complement components

The major components of complement, receptors, and activation fragments are found in human milk, and the presence of complement components in maternal breast milk has been documented for decades in multiple mammalian species besides humans.^137,138^ Complement components are present at relatively higher concentrations in colostrum and transitional milk compared to their levels in mature milk.^139,140^ It has been proposed that complement components participate in bacteriolysis, neutralization of pathogens, and immunoconglutination, as well as enhancing phagocytosis in the infant gut.^91^ Moreover, previous *in vitro* studies showed that complement components present in maternal milk exhibited bactericidal/bacteriostatic ability against selective pathogens including *Escherichia coli* and *Helicobacter pylori*.^141,142^ It has been long assumed that complement components in maternal milk should make important contributions to mucosal immunity in the infant gut and to preventing the infant from enteric infections; however, the pathophysiological significance, especially the underlying mechanisms, has been poorly understood until recent insights from our studies into the crucial role of maternal milk-supplied complement system in shaping the infant gut microbiota.^143^

Our studies, utilizing a comprehensive set of mouse breeding strategies, demonstrate that complement components present in maternal milk selectively kills *Staphylococcus* bacteria in the gut commensal microbiota of suckling pups in a complement component 1 (C1) dependent but antibody independent pathway, thus modulating the composition of infant gut microbiota.^143^ Indeed, the weanling mice fostered by dams deficient in complement components in breast milk harbor distinct gut microbiota compositions, characterized with the elevated abundance of *Staphylococcus*, *Coprococcus*, *Streptococcus*, *Lactobacillus*, and *Ruminococcus*, as well as reduced levels of *Adlercreutzia*, *Allobaculum*, *Bifidobacterium*, *Turicibacter*, and *Anaerovorax*, in comparison to the suckling animals breastfed with complement-sufficient maternal milk. Such alterations in the early-life gut microbiota harbor important pathophysiological significance for weanling mice, resulting in a failure to thrive and death upon challenge with enteric infection with *Citrobacter rodentium*. Maternal milk-supplied complement components exhibit no direct bactericidal/bacteriostatic effects on the pathogen *Citrobacter rodentium*; instead, they possess an evolutionally conserved capacity to eliminate selective commensal microbes, thus shaping the infant gut microbiota during early life. Of note, the infant gut microbiota shaped by complement components supplied in maternal milk adds a new layer to the protective mechanisms offered by breastfeeding to promote infant health and defend against environmental pathogens.

## 9. Conclusions and perspectives

**Figure 1.**
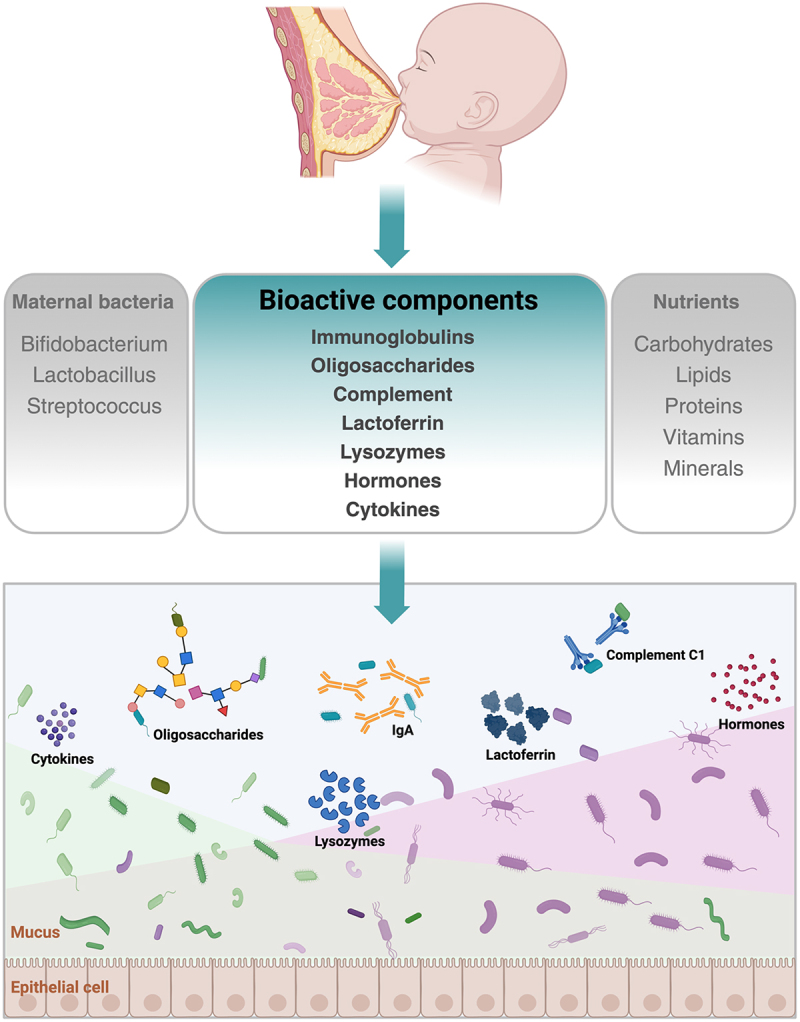
Bioactive components in maternal milk shape the gut microbiota compositions during infancy.

The assembly of gut microbiota in early life is an orchestrated process that results in specialized microbial ecosystems with longstanding consequences for host health. During infancy, a critical period for establishment of the gut microbiota, gut microbial compositions change rapidly and dynamically until reaching homeostasis.^144^ This complex process of colonization to maturity of the microbiota community in the infant gut is affected by diverse genetic and environmental factors,^145,146^ of which breastfeeding has been widely appreciated as a preponderant determinant.^147,148^ Indeed, the association between breastfeeding and establishment of the infant gut microbiota, in physiological and pathological settings, has remained an active area of investigation. In particular, it has been well documented that the essential macronutrients and micronutrients offered by breastfeeding stimulate the most adequate microbial growth and development in the infant gut.^16,23,24^ Emerging evidence indicates that a rich and diverse bacterial community present in maternal milk, *i.e*., maternal milk microbiota, could be a unique source for microbial colonization in the infant gut.^26–28^ The relationships between compositions of the infant gut microbiota and some bioactive components have been extensively studied and fairly well understood in the past decades (Figure 1). Interestingly, the potential mechanisms through which other bioactive components present in maternal milk contribute to shaping the gut bacterial communities during infancy, which had long been speculated, have started to emerge, in particular complement components present in maternal milk as recently revealed by our own studies.^143^ These findings support the notion that while there is a remarkable evolutionary adaptation to select bacterial species for infant gut microbiota, maternal milk has evolved to indirectly promote infant health.^7^

While studies have established the role of critical components present in maternal milk in shaping the infant gut microbiota, the underlying mechanisms by which breastfeeding may confer this relationship remain poorly understood. It is noteworthy that bidirectional communications between microbial colonization and immune development have been proposed, in which not only gut microbiota affect the development of the immune system but also the host immune system influences the development of the gut microbiota.^149^ Hence, the various immunological components present in maternal milk, such as cytokines and hormones, may not act directly on the gut microbiota, rather execute crucial functions in immune development that could influence the infant gut microbiota indirectly.^150^ There are needs to conduct deeper sequencing and comprehensive metabolite profiling of the infant gut microbiota to gain better insights into microbial functions, and to understand whether and how bioactive components present in maternal milk act synergistically to shape the infant gut microbiota. A better understanding of the relationship between breastfeeding and the evolving infant gut microbiota during early life, in particular how various bioactive components in maternal milk impact colonization and establishment of the gut microbiota during infancy, will facilitate rational development of microbiota-tailored interventions to improve human health.
